# VSMCs and the immune microenvironment: a multidimensional regulatory network driving vascular injury and repair

**DOI:** 10.3389/fimmu.2026.1844567

**Published:** 2026-06-05

**Authors:** Yuxin Wei, Yuanpeng Liao, Cong Zhang, Jiawei Guo

**Affiliations:** 1 Department of Vascular and Endovascular Surgery, The First Affiliated Hospital of Yangtze University, Jingzhou, China; 2 Department of Pharmacology, School of Medicine, Yangtze University, Jingzhou, China

**Keywords:** cardiovascular diseases, immune microenvironment, inflammatory response, phenotypic remodeling, vascular smooth muscle cells

## Abstract

The dynamic interplay between vascular smooth muscle cells (VSMCs) and the immune microenvironment governs vascular repair and disease progression. This review systematically elucidates this multidimensional regulatory network. We primarily dissect the molecular mechanisms driving pathological VSMC remodeling, encompassing inflammatory signaling, regulated cell death, senescence-associated DNA damage, and organelle dysfunction. Furthermore, we delineate the bidirectional crosstalk connecting VSMCs with the extracellular matrix (ECM) and tissue-infiltrating leukocytes, notably macrophages, T cells, and neutrophils. Subsequently, we evaluate how this network dictates disease-specific pathophysiology across various cardiovascular diseases. Specifically, we analyze metabolic reprogramming and epigenetic regulation in atherosclerosis (AS), mechanosensitive inflammation-contraction coupling in hypertension, and kinase-driven organelle stress in aortic aneurysms and dissection. Moreover, we examine the secretory profile of VSMCs in pulmonary arterial hypertension (PAH) and their contribution to microvascular dysfunction in arteritis and cardiomyopathy. Ultimately, we highlight emerging therapeutic strategies targeting VSMC-immune interactions and discuss future translational prospects.

## 1 Introduction

Cardiovascular diseases remain the leading global cause of mortality, fundamentally driven by pathological vascular wall remodeling. As the predominant cellular constituent of the medial layer, VSMCs maintain vascular tone and structural integrity while exhibiting profound phenotypic plasticity. Under homeostatic conditions, VSMCs maintain a differentiated contractile state. However, pathological stimuli provoke profound dedifferentiation into a synthetic, highly proliferative, and migratory phenotype. Consequently, this phenotypic switching constitutes the core response to vascular injury. Nevertheless, the multidimensional regulatory mechanisms governing this transition, particularly the intricate crosstalk with the local cellular microenvironment, remain incompletely defined.

Emerging evidence establishes that VSMCs transcend their traditional role as passive structural effectors, functioning instead as active immunomodulatory cells. Within the vascular niche, VSMCs continuously integrate mechanical stress, metabolic cues, and inflammatory signals. Accordingly, they execute robust intracellular responses involving inflammatory cascade activation, DNA damage sensing, and organelle homeostatic regulation. Furthermore, VSMCs engage in reciprocal signaling with the ECM and infiltrating immune cells. Ultimately, this intricate intercellular communication network dictates cell fate and drives the pathogenesis of diverse vascular pathologies, notably AS, hypertension, aortic aneurysm and dissection, PAH, and vasculitis.

Therefore, deciphering the dynamic interface between VSMCs and the immune microenvironment is paramount for discovering novel therapeutic vulnerabilities. This review systematically evaluates the multidimensional regulatory networks orchestrating VSMC-mediated vascular injury and repair. Initially, we define the molecular drivers of VSMC phenotypic remodeling, emphasizing the contributions of cellular senescence and organelle stress. Subsequently, we detail the complex communication cascades bridging VSMCs, the ECM, and diverse immune populations. We then contextualize these mechanisms within specific clinical paradigms, elucidating metabolic reprogramming in AS, inflammation-contraction coupling in hypertension, and autocrine/paracrine signaling in PAH. Finally, we evaluate emerging therapeutic interventions targeting VSMC immunobiology, providing a theoretical framework to advance precision cardiovascular medicine.

## 2 Mechanisms

### 2.1 Pathological remodeling mechanisms and multidimensional regulatory mechanisms of VSMCs

**Figure 1:**
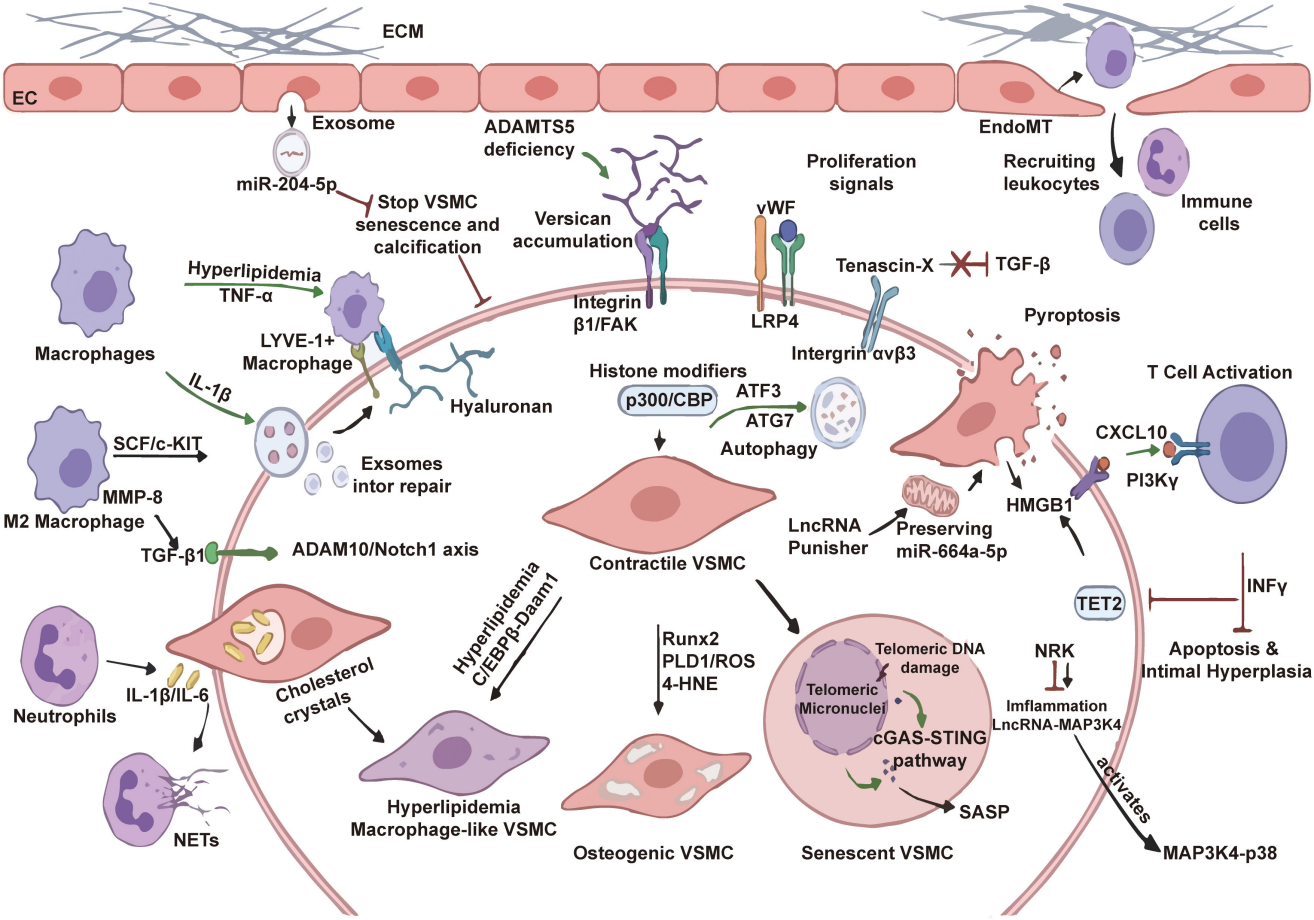
VSMC-immune microenvironment network in vascular remodeling. Pathologically, contractile VSMCs undergo multidirectional phenotypic switching to orchestrate inflammation (1).Phenotypic reprogramming: hyperlipidemia and oxidative stress drive macrophage-like, osteogenic, and senescent VSMC transitions, thereby activating DNA damage-induced cGAS-STING signaling, SASP secretion, and Caspase-3/GSDME-dependent pyroptosis (2).Endothelial-ECM niche: EC-derived exosomal miR-204-5p attenuates senescence, whereas EndoMT provokes leukocyte recruitment. Concurrently, ECM remodeling and vWF interactions dictate VSMC proliferation and osteogenesis (3).Myeloid crosstalk: macrophage-derived IL-1β/TNF-α/MMP-8 exacerbate neointimal hyperplasia; conversely, LYVE-1+ and M2 macrophages orchestrate vascular repair. Furthermore, cholesterol-laden VSMCs recruit neutrophils, triggering NET formation (4). Lymphoid-epigenetic control: VSMC-derived CXCL10 activates T cells, reciprocally eliciting IFNγ secretion to suppress TET2 and precipitate apoptosis. Concurrently, intracellular kinases and lncRNAs fine-tune this cascade. VSMC, Vascular smooth muscle cell; ECM, Extracellular matrix; SASP, Senescence-associated secretory phenotype; EC, Endothelial cell; EndoMT, Endothelial-to-mesenchymal transition; vWF, von Willebrand factor; NETs, Neutrophil extracellular traps; ROS, Reactive oxygen species.

Under pathological conditions, VSMCs transcend their contractile role to orchestrate inflammation, calcification, and vascular remodeling via immunomimicry and osteogenic transdifferentiation. Hyperlipidemia drives VSMCs into a phagocytic, macrophage-like state via the C/EBPβ-Daam1 axis (**Figure 1**) (1). Concurrently, Runx2 promotes osteogenic differentiation and inflammatory cytokine expression, establishing a calcification-inflammation positive feedback loop (2). Oxidative stress aggressively exacerbates these phenotypic shifts. Specifically, phospholipase D1 accelerates VSMC proliferation via reactive oxygen species (ROS) generation (3), whereas 4-HNE-mediated Runx2 modification promotes an osteo-inflammatory phenotype (4). In contrast, Physalin B exerts vasoprotective effects by clearing ROS via Nrf2 pathway activation (5). Furthermore, epigenetic regulators dictate cellular fate; histone acetyltransferases p300 and CBP reciprocally govern contractile gene expression (6). Similarly, thioxanthine-interacting protein inhibits osteochondral conversion (7), while microRNA-26b depletion accelerates aortic calcification by derepressing osteogenic targets (8). Regarding mechanotransduction, the loss of YAP/TAZ triggers cGAS-STING-dependent autoinflammation, directly driving vascular remodeling (9). Crucially, the trajectory of these phenotypic shifts is fundamentally governed by VSMC embryonic lineage (10). The vasculature is a mosaic structure comprising VSMCs from distinct origins, prominently the neural crest in the aortic arch and the lateral plate mesoderm in the descending aorta. These lineage-specific cells retain persistent epigenetic memories that drive divergent responses to signaling cascades such as TGF-β. While TGF-β signaling typically preserves the contractile phenotype in mesoderm-derived VSMCs, neural crest-derived cells are intrinsically predisposed to TGF-β-mediated pro-inflammatory and osteogenic transdifferentiation (11). Recognizing this profound lineage heterogeneity is essential, as it elegantly explains the spatial predilection of vascular diseases and intrinsic variations in immunomodulatory plasticity (12).

### 2.2 Inflammatory networks, senescence, and cell death

Vascular wall inflammation is intricately regulated by kinase networks, non-coding RNAs, and programmed cell death. For instance, Nik-related kinase protects against intimal hyperplasia by dampening local inflammation (13). Conversely, LncRNA-MAP3K4 amplifies inflammatory cascades through trans-regulation of the MAP3K4-p38 MAPK pathway, exacerbating vascular injury (14). Under severe stress, the Caspase-3/GSDME axis executes VSMC pyroptosis, releasing potent pro-inflammatory mediators like HMGB1 to aggravate medial calcification(**Figure 1**) (15). Furthermore, exogenous insults, including SARS-CoV-2 infection, activate the IL18/IL18R1/HIF-1 axis, highlighting the synergistic detriment of hypoxia and inflammation (16). Crucially, senescence-associated sterile inflammation profoundly activates local immunity. Telomeric DNA damage generates micronuclei, which activate the cytoplasmic cGAS-STING pathway to induce the senescence-associated secretory phenotype (SASP), sequentially recruiting immune cells(**Figure 1**) (17). Oxidative stress similarly triggers SASP, secreting pro-inflammatory factors that recruit leukocytes and abnormally stimulate adjacent VSMC proliferation (18). Maintaining organelle homeostasis is essential to resist this inflammatory senescence. The transcription factor ATF3 delays senescence via ATG7-mediated autophagy, although m6A modification compromises its stability (19). Alternatively, the LncRNA Punisher preserves mitochondrial integrity and prevents apoptosis by targeting the miR-664a-5p/OPA1 axis (20). Ultimately, systemic endothelial dysfunction-induced chronic oxidative stress establishes the pathological microenvironment driving VSMC functional decline (21).

### 2.3 Crosstalk between the ECM and endothelial nichet

ECM dynamics strictly govern VSMC function and plaque stability. Deficiencies in the ECM protease ADAMTS5 cause versican accumulation, which triggers integrin β1/FAK signaling and subsequent VSMC osteogenesis (22). In contrast, sclerins maintain cellular homeostasis and limit calcification by antagonizing the Wnt/β-catenin cascade (23). Beyond hemostasis, von Willebrand factor promotes VSMC proliferation by activating LRP4-dependent integrin αvβ3 signaling (24). Conversely, VSMC-derived Tenascin-X fosters pathological proliferation by suppressing TGF-β signaling; accordingly, Tenascin-X ablation restores TGF-β-mediated anti-proliferative effects to inhibit vascular remodeling (25). Furthermore, elevated secreted phosphoprotein 1 strongly correlates with coronary plaque vulnerability (26). The endothelial barrier actively modulates VSMC plasticity; healthy endothelial cells transfer exosomal miR-204-5p to inhibit VSMC senescence and calcification(**Figure 1**) (27). However, during AS, endothelial-to-mesenchymal transition amplifies leukocyte recruitment, indirectly exacerbating VSMC pathology (28).

### 2.4 Bidirectional communication between VSMCs and immune cells

VSMCs engage in profound, bidirectional crosstalk with tissue-infiltrating leukocytes(**Figure 1**). Regarding T cells, VSMC-derived CXCL10 impedes endothelial repair by activating T cells via a PI3Kγ-dependent pathway (29). Subsequently, activated T cells secrete IFNγ to suppress TET2 in VSMCs, precipitating apoptosis and compensatory intimal hyperplasia (30). Moreover, extracellular vesicles from senescent VSMCs modulate T-cell and monocyte function, fundamentally remodeling the local immune niche (31). Pathologically, T-cell-mediated hyperinflammation directly drives VSMC proliferation and arteriovenous fistula stenosis (32).

Simultaneously, macrophage-derived cytokines, notably IL-1β and TNF-α, aggressively drive VSMC synthetic and osteogenic switching (33). Macrophage-specific Epsins promote VSMC foam cell formation, and their targeted deletion significantly reduces plaque burden (34). Furthermore, paracrine signals from Drp1-mediated mitochondrial fission in pro-inflammatory macrophages accelerate intimal thickening (35). Although M2 macrophage-derived exosomes facilitate VSMC repair via the SCF/c-KIT pathway, they concurrently risk exacerbating restenosis through hyperproliferation (36). Notably, CD163+ macrophages present a clinical paradox by inhibiting calcification while simultaneously destabilizing plaques (37). Conversely, a distinct LYVE-1+ macrophage subset preserves vascular homeostasis by binding VSMC-derived hyaluronan (38). Additionally, macrophage-secreted MMP-8 exhibits non-proteolytic function by cleaving TGF-β1, which activates ADAM10/Notch1 signaling to drive adventitial progenitor differentiation into neointimal VSMCs (39).

Finally, VSMCs actively dictate neutrophil chemotaxis. Following cholesterol crystal phagocytosis, VSMCs release IL-1β and IL-6 to recruit neutrophils and trigger neutrophil extracellular trap (NET) formation, establishing a destructive inflammatory cycle within the vascular wall (40).

### 2.5 Single-cell insights into the functional dichotomy of VSMC subpopulations

The advent of single-cell RNA sequencing has revolutionized our understanding of VSMC diversity, transcending the binary contractile versus synthetic model (12). Recent single-cell RNA sequencing landscapes have identified several functionally distinct subpopulations that exert opposing influences on vascular pathology. On one hand, VSMCs can transdifferentiate into fibromyocytes or myofibroblast-like cells, which are characterized by high extracellular matrix production and are predominantly localized in the protective fibrous cap, thereby promoting plaque stability and disease resolution (12). Conversely, a subset of VSMCs adopts a macrophage-like or pro-inflammatory phenotype, characterized by the loss of contractile markers and the acquisition of phagocytic and cytokine-secreting capabilities, which aggressively drive lipid accumulation and chronic inflammation. Furthermore, single-cell RNA sequencing has identified an intermediate multipotent state, often termed SMC-derived intermediate cells, which serve as a transitional reservoir capable of bifurcating into either protective or detrimental fates depending on the local microenvironment (41). This single-cell resolution reveals that VSMC-driven remodeling is not a monolithic process but a fine-tuned balance between competitive subpopulations, where the predominance of specific clusters dictates the trajectory toward disease progression or resolution (10).

## 3 Pathophysiological role in cardiovascular disease

### 3.1 Atherosclerosis

#### 3.1.1 Phenotypic characteristics, cellular metabolism and genetic regulation of VSMCs

AS is fundamentally a VSMC-driven pathology characterized by oligoclonal expansion rather than stochastic cellular proliferation. Clonal VSMCs profoundly dedifferentiate, expressing macrophage markers notably including Lgals3 and Mac2. This transdifferentiation is strictly bidirectional. Initially, lipid infiltration triggers VSMC foam cell formation (42, 43). Subsequently, macrophage-derived Fortilin prevents their reversion to a contractile state, perpetuating local inflammation (44). Crucially, VSMC transdifferentiation generates approximately 50% of the total plaque foam cell population (45, 46), fostering pronounced intra-plaque phenotypic heterogeneity driven by clonal VSMC expansion. Specifically, VSMCs within the calcified core upregulate osteogenic genes, including POSTN and SPP1, whereas those in adjacent zones adopt a TNF-α-driven inflammatory phenotype (47). Furthermore, diseased VSMCs aggressively upregulate the CD47 anti-phagocytic signal to evade macrophage clearance, thereby precipitating secondary necrosis and necrotic core expansion (45, 48).

Mechanistically, metabolic reprogramming and impaired autophagy precipitate VSMC foaming. VSMCs actively augment *de novo* lipogenesis via FASN and TMEM41B upregulation, while simultaneously exhibiting deficient lipid degradation due to downregulated lysosomal acid lipase (49–51). Concurrently, TRIM13-mediated ubiquitination degrades the nuclear receptors LXRα and LXRβ, suppressing cholesterol efflux and exacerbating lipid retention (52).Additionally, deficiencies in NFAT5 (53) or ADAM17 under hyperlipidemic conditions strongly induce lipid phagocytosis and macrophage-like transformation (54). While basal autophagy preserves VSMC homeostasis, CARM depletion downregulates CSNK1A1, inhibiting the AKT/ATG7 axis to arrest autophagic flux and accelerate foam cell conversion (55). Similarly, P2RY12 activation impedes cholesterol clearance by suppressing PI3K/AKT/mTOR-dependent autophagy (56). Crucially, O-GlcNAc transferase deficiency abolishes protective O-GlcNAcylation, sensitizing VSMCs to oxidized low-density lipoproteins. Consequently, this triggers massive cellular demise through the simultaneous activation of apoptosis, necroptosis, and pyroptosis, severely accelerating necrotic core formation (57).

Transcriptional and epigenetic networks tightly govern VSMC plasticity. Endoplasmic reticulum stress-induced KLF4 upregulation acts as a primary dedifferentiation catalyst, a process amplified by PIAS3 deficiency-mediated reduction in KLF4 SUMOylation (58, 59). Moreover, transcription factors including HOXA1, Slug, and c-Fos drive pro-inflammatory and foam cell phenotypes via the NF-κB, PDGF-BB/ERK, and LOX-1 cascades, respectively (60, 61). Chromatin accessibility sequencing indicates that ATF3 strictly initiates VSMC transition into macrophage-like pathogenic states (62). Furthermore, post-transcriptional dysregulation, including ADAR1-deficient aberrant RNA editing (63) and defective splicing of Abi1 and STAT3 mediated by MBNL1 and PCBP1, robustly drives phenotypic switching (64, 65). Regarding non-coding RNAs, the lncRNA INKILN accelerates inflammation (66), whereas Let-7d-5p provides atheroprotection (67). Conversely, intrinsic protective pathways antagonize these maladaptive changes. Specifically, LKB1-dependent SIRT6 activation attenuates foaming by repressing LOX-1 (68, 69), PGC1α preserves the contractile phenotype (70), and the histone methyltransferase DOT1L restricts NF-κB-driven excessive activation and monocyte recruitment (71).

#### 3.1.2 VSMC-mediated inflammatory microenvironment, cell-cell interactions and ageing mechanisms

VSMCs actively orchestrate the vascular inflammatory microenvironment. They constitute the predominant source of IL-1β in early-stage coronary lesions, and targeted ablation of VSMC IL-1 receptors significantly attenuates plaque burden (72, 73). Nevertheless, IL-1 signaling exhibits highly pleiotropic effects. While thrombin-cleaved IL-1α drives inflammation, it concurrently stimulates VSMC proliferation and collagen deposition to stabilize the fibrous cap (74). However, the current literature presents critical contradictory findings regarding this pathway, highlighting a major unresolved paradox concerning the dual role of IL-1β. While canonically viewed as a potent pro-inflammatory driver of early atherogenesis, recent advanced lineage-tracing studies reveal that IL-1β is paradoxically essential for maintaining VSMC investment within the fibrous cap and promoting plaque stability in late-stage lesions (75). Consequently, while indiscriminate IL-1β blockade effectively suppresses systemic inflammation, it severely risks precipitating plaque rupture in advanced disease by impairing VSMC-mediated extracellular matrix synthesis and cap repair (12). Reconciling these highly stage-dependent and opposing functions of IL-1β remains a critical frontier in cardiovascular immunology (10). Therefore, indiscriminate IL-1 blockade risks precipitating plaque rupture despite suppressing systemic inflammation. Environmentally, NLRP3 inflammasome assembly, MyD88 cascade activation, and the acid-sensing receptor Tdag8 collectively exacerbate pathological VSMC proliferation and migration (76–78). Similarly, internalized environmental pollutants, notably titanium dioxide nanosheets, induce profound VSMC foaming via upregulation of the non-canonical NF-κB subunit NFKB2 (79). Furthermore, the microbial metabolite TMAO directly accelerates phenotypic switching and medial inflammation (80). Notably, hypoxia represses VSMC-derived EPAC1 secretion, establishing it as a highly sensitive biomarker for plaque hypoxic severity (81). In contrast, vitamin D confers atheroprotection by antagonizing the JNK-TLR4 axis, thereby downregulating scavenger receptors like CD36 while upregulating ABCA1, ABCG1, and LXR-α-mediated cholesterol efflux (82).

Extensive bidirectional crosstalk exists between VSMCs and macrophages. Macrophage-derived IL-1β and miR-19b-3p-enriched extracellular vesicles aggressively induce VSMC pro-inflammatory transformation (83, 84). Conversely, M2 macrophage-derived exosomal miR-7683-3p facilitates VSMC cholesterol efflux to attenuate foaming (85). Reciprocally, VSMCs secrete pro-atherogenic matrix proteins, including SVEP1 and fibronectin EDA, to amplify macrophage chemotaxis and local inflammation (86, 87). Furthermore, senescence profoundly exacerbates these interactions. Senescent VSMCs upregulate dipeptidyl peptidase 4 to cleave coagulation factor X and activate the PAR2 receptor, thereby igniting intense vascular inflammation (88). Concurrently, the plaque-enriched lncRNA JPX orchestrates structural chromatin remodeling to activate senescence-associated genes, directly driving VSMC aging and atheroprogression (89).

Collectively, while these intricate mechanisms—from early phenotypic switching to terminal senescence—highlight the multifaceted role of VSMCs in atherogenesis, critical gaps remain. A major translational bottleneck is the unresolved IL-1β paradox; current literature lacks consensus on how to therapeutically target the early pro-inflammatory IL-1β cascade without compromising its essential role in fibrous cap maintenance during advanced stages. Furthermore, future research must delineate the precise spatiotemporal triggers that dictate whether a dedifferentiated VSMC adopts a macrophage-like, osteogenic, or alternative pathogenic phenotype within the highly heterogeneous plaque microenvironment.

### 3.2 Hypertension and vascular remodeling

#### 3.2.1 Immunological properties and inflammatory regulation of VSMCs

**Figure 2:**
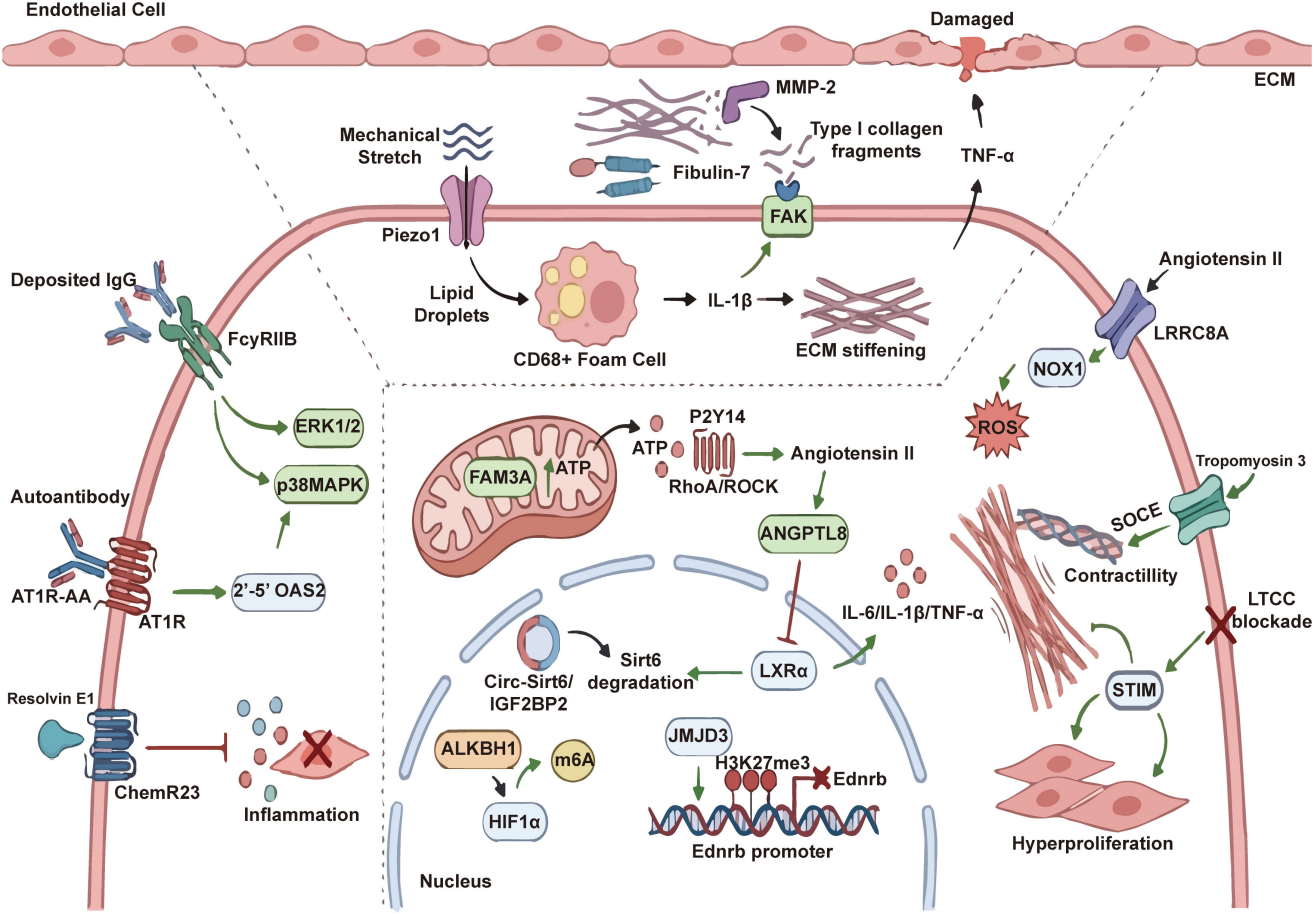
VSMC regulatory network in hypertensive vascular remodeling. Phenotypically switched VSMCs orchestrate vascular inflammation (1). Immune receptors: IgG-FcγRIIB interactions activate ERK1/2-p38MAPK cascades, and AT1R-AA stimulates 2’-5’ OAS2, whereas Resolvin E1-ChemR23 signaling resolves inflammation (2). Mechano-ECM niche: stretch-activated Piezo1 drives CD68+ foam cell transition. Concurrently, MMP-2-cleaved collagen stimulates FAK, while VSMC-derived IL-1β/TNF-α provoke ECM stiffening and endothelial dysfunction (3). Metabolic-epigenetic reprogramming: FAM3A-amplified ATP activates P2Y14/RhoA/ROCK signaling, and angiotensin II-induced ANGPTL8 represses LXRα to precipitate cytokine release. Epigenetically, Circ-Sirt6 depletion accelerates Sirt6 degradation, ALKBH1 demethylates HIF1α m6A, and JMJD3 deficiency accumulates repressive H3K27me3 silencing Ednrb (4). calcium-ion dynamics: angiotensin II-activated LRRC8A stimulates NOX1-dependent ROS, and tropomyosin 3-SOCE synergy augments contractility. Paradoxically, chronic LTCC blockade induces maladaptive hyperproliferation via compensatory STIM activation. VSMC, Vascular smooth muscle cell; ECM, Extracellular matrix; AT1R, Angiotensin II type 1 receptor; FAK, Focal adhesion kinase; ROS, Reactive oxygen species; SOCE, Store-operated calcium entry; LTCC, L-type calcium channel; STIM, Stromal interaction molecule.

Rather than acting as passive targets, VSMCs function as active immune modulators capable of directly recognizing autoantibodies and lipid mediators. In hypertensive states, VSMCs significantly upregulate the low-affinity IgG receptor FcγRIIB. The subsequent binding of deposited IgG activates intracellular ERK1/2 and p38MAPK cascades independently of classical leukocytes, directly driving VSMC hyperproliferation and inflammation(**Figure 2**) (90). Furthermore, the hypertension-associated autoantibody AT1R-AA directly agonizes the angiotensin II type 1 receptor on VSMCs. This uniquely activates the innate immune sensor 2’-5’ oligoadenylate synthetase 2 to precipitate phenotypic switching (91). Nevertheless, VSMCs retain robust intrinsic resolving capabilities. Hypertensive stress compensatorily upregulates the surface resolution receptor ChemR23, which binds Resolvin E1 to actively suppress local inflammation (92).

#### 3.2.2 Mechanically and microenvironmentally driven phenotypic transformation

Biomechanical stress and ECM remodeling fundamentally dictate VSMC transdifferentiation. Remarkably, hypertensive mechanical stretch alone, devoid of biochemical cues, suffices to induce intracellular lipid droplet accumulation via the mechanosensitive Piezo1 channel. This triggers a direct transition into CD68-positive, non-contractile foam cells, proving that physical forces independently drive immunophenotypic switching (93). Within the microenvironment, matrix metalloproteinase-2-mediated type I collagen fragments activate focal adhesion kinase in VSMCs, seamlessly coupling ECM degradation to hyperproliferative intracellular signaling (94). Additionally, structural proteins like fibulin-7 critically regulate this dedifferentiation (95). Consequently, transformed VSMCs secrete abundant IL-1β, creating a vicious cycle of progressive ECM degradation and severe vascular stiffening (96). Concurrently, paracrine TNF-α secretion from these VSMCs profoundly impairs adjacent endothelial function (97).

#### 3.2.3 Metabolic reprogramming and epigenetic regulation

Energy dysmetabolism and epigenetic dysregulation synergistically sustain the hypertensive VSMC phenotype. Metabolically, Angiotensin II massively upregulates angiopoietin-like protein 8, which represses liver X receptor alpha to perturb lipid metabolism and aggressively stimulate IL-6, IL-1β, and TNF-α secretion, exacerbating remodeling (98). Furthermore, elevated mitochondrial FAM3A amplifies ATP extracellular release. This activates the P2Y14 receptor and RhoA/ROCK cascades, triggering severe medial inflammation and hypercontractility, a phenotype entirely reversed by FAM3A ablation (99). Epigenetically, hypertension dismantles critical homeostatic networks. Typically, Circ-Sirt6 recruits IGF2BP2 to stabilize Sirt6 mRNA; however, hypertensive stimuli profoundly downregulate Circ-Sirt6, accelerating Sirt6 degradation and unleashing unchecked VSMC proliferation (100). Concurrently, the demethylase ALKBH1 is pathologically upregulated, erasing N6-methyladenosine marks from the HIF1α gene to hyperactivate hypoxic transcription (101). Simultaneously, hypertension suppresses the protective histone demethylase JMJD3, leading to repressive H3K27me3 accumulation at the Ednrb promoter. This extinguishes the expression of the vasoprotective endothelin receptor B, permanently amplifying VSMC sensitivity to vasoconstrictors (102).

#### 3.2.4 Inflammation-contraction coupling via ion channels and calcium signaling

Calcium transients not only govern excitation-contraction coupling but fundamentally dictate inflammatory gene transcription. Emerging evidence highlights a critical synergy between ion channels and cytoskeletal architecture in perpetuating VSMC pathology. During Angiotensin II-induced hypertension, the volume-regulated anion channel LRRC8A drives massive vascular oxidative stress and endothelial dysfunction by upregulating NADPH oxidase 1 (103). Furthermore, the cytoskeletal protein tropomyosin 3 synergizes with store-operated calcium entry to mutually augment VSMC contractility and pathological proliferation (104). Consequently, monotherapy targeting isolated channels proves severely limited; chronic L-type calcium channel blockade paradoxically triggers compensatory calcium network remodeling. This activates STIM proteins, ultimately driving maladaptive VSMC hyperproliferation and migration (105).

This paradoxical response to monotherapy underscores a fundamental limitation in current therapeutic strategies for hypertensive remodeling. Because targeting isolated channels triggers compensatory cellular networks, a major outstanding question is how to develop synergistic interventions that simultaneously target upstream physical mechanosensors such as Piezo1 and their downstream inflammatory cascades. Future studies must also decode how mechanical stretch and autoantibody signaling cross-talk at the epigenetic level to sustain this intractable VSMC hyperproliferation.

### 3.3 Aortic dissection and aortic aneurysm

The pathogenesis of aortic dissection (AD) and abdominal aortic aneurysm (AAA) is driven by complex interactions between hemodynamic perturbations and immune-inflammatory cascades. Crucially, VSMCs dysfunction, depletion, and phenotypic reprogramming constitute the core pathological mechanisms (106). Furthermore, intrinsic VSMC heterogeneity and plasticity dictate subpopulation-specific responses to inflammatory stimuli, providing a cellular basis for localized disease onset (107). The pathogenesis of aortic dissection and abdominal aortic aneurysm is driven by complex interactions between hemodynamic perturbations and immune-inflammatory cascades. Crucially, VSMC dysfunction, depletion, and phenotypic reprogramming constitute the core pathological mechanisms 106. Furthermore, intrinsic VSMC heterogeneity and plasticity dictate subpopulation-specific responses to inflammatory stimuli, providing a cellular basis for localized disease onset 107. To distinguish between shared and disease-specific roles, it is essential to note that mechanisms such as KLF4, NF-κB, cGAS-STING, and SIRT6, which primarily govern lipid-rich foam cell transdifferentiation and plaque remodeling in atherosclerosis, are redirected in aneurysmal disease to drive medial thinning and extracellular matrix degradation. Specifically, these pathways in the aneurysmal aorta converge to accelerate proteolytic destruction and mechanical wall failure rather than plaque-centric inflammation.

#### 3.3.1 External environmental factors and metabolism

**Figure 3:**
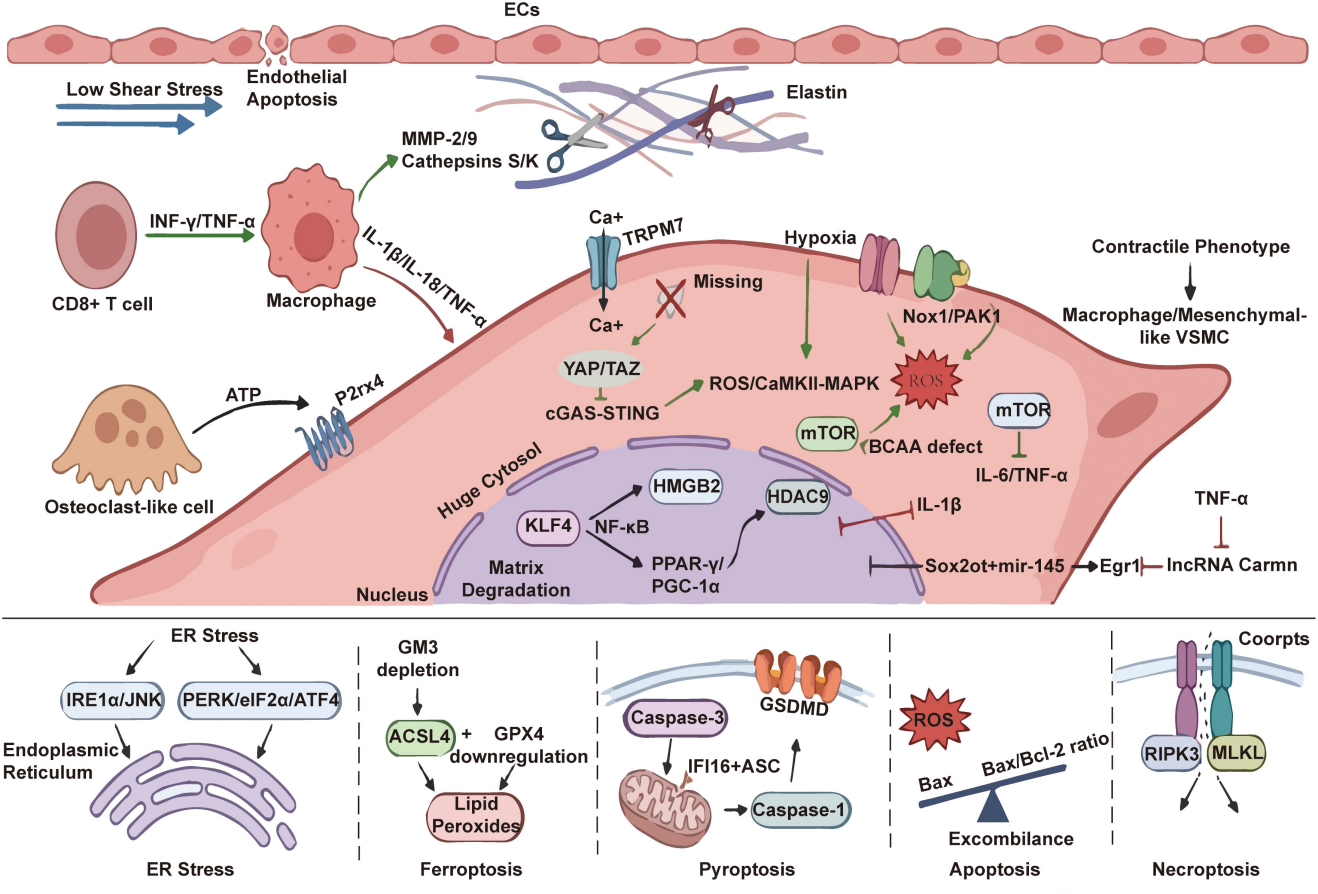
Pathogenesis of AD and aneurysm (1). Immune-ECM niche: low shear stress induces endothelial apoptosis. Concurrently, CD8+ T cell-derived IFN-γ/TNF-α activate macrophages, precipitating VSMC-targeting cytokine release and MMP-2/9/Cathepsin S/K-mediated elastin degradation. Furthermore, osteoclast-like cells secrete ATP to activate VSMC P2rx4 receptors (2). Phenotypic reprogramming: VSMCs adopt macrophage/mesenchymal-like phenotypes via TRPM7-dependent Ca2+ influx, YAP/TAZ deficiency-induced cGAS-STING activation, and hypoxia-triggered ROS/CaMKII-MAPK cascades. Additionally, BCAA-sensing mTOR and Nox1/PAK1 amplify cytokine and ROS generation (3). Epigenetic-transcriptional control: KLF4 dictates matrix degradation, while HMGB2 activates NF-κB and PPAR-γ/PGC-1α axes. Epigenetically, HDAC9 deacetylates histones silencing contractile genes, whereas SIRT6 represses IL-1β. Moreover, Sox2ot-miR-145 interaction upregulates Egr1, and macrophage-derived TNF-α silences lncRNA Carmn (4). Stress and lethality: ER stress propagates via IRE1α/JNK and PERK/eIF2α/ATF4 pathways. Consequently, VSMCs execute programmed death via ACSL4-driven lipid peroxidation upon GM3/GPX4 depletion, GSDME/GSDMD-mediated pyroptosis, ROS-induced Bax/Bcl-2 skewing, and RIPK3/MLKL-dependent membrane disruption. AD, Aortic dissection; AAA, Abdominal aortic aneurysm; VSMC, Vascular smooth muscle cell; ECM, Extracellular matrix; ROS, Reactive oxygen species; BCAA, Branched-chain amino acid; ER stress, Endoplasmic reticulum stress.

Biomechanical and metabolic perturbations critically initiate VSMC inflammatory transitions. Disturbed hemodynamics, notably low shear stress at vascular bifurcations, potently activate NF-κB-driven immune cascades and upregulate Caspase-3 and Caspase-12. This sequentially precipitates endothelial apoptosis and disrupts mural homeostasis(**Figure 3**) (108). Concurrently, defective mechanotransduction exacerbates this injury. Specifically, YAP/TAZ mechanosensor deficiency abolishes the contractile phenotype and spontaneously triggers cGAS-STING-dependent sterile inflammation (109). Environmentally, chronic intermittent hypoxia induces VSMC apoptosis and transdifferentiation via the ROS/CaMKII-MAPK signaling axis (110). Additionally, hypoxia suppresses the protective molecule RECK, unleashing matrix metalloproteinases to irreversibly degrade elastin and compromise vascular integrity (111). Similarly, exposure to the plasticizer DEHP provokes oxidative ROS/NF-κB activation to precipitate AAA formation (112). Conversely, the tricarboxylic acid cycle intermediate alpha-ketoglutarate confers protection by attenuating oxidative stress-induced VSMC apoptosis and inflammatory infiltration (113).

#### 3.3.2 Molecular regulation of VSMC phenotypic reprogramming

During aneurysm pathogenesis, inflammatory cues orchestrate VSMC transdifferentiation into macrophage-like or mesenchymal-like states. This reprogramming is masterminded by the transcription factor KLF4. Specifically, TRPM7-mediated calcium influx activates KLF4 to drive matrix degradation and pathological remodeling, whereas targeted KLF4 downregulation halts aneurysmal expansion (114, 115). Conversely, FAM3A preserves the differentiated phenotype by modulating KLF4 ubiquitination (116). Furthermore, single-cell transcriptomics confirms that aneurysmal VSMCs adopt a mesenchymal signature, orchestrating immune crosstalk via TNF-TNFRSF1A signaling (117), with discrete subpopulations acting as primary inflammatory hubs (118). Additionally, Fcγ receptor activation allows VSMCs to bind immune complexes and secrete MMP-2 (119), while S100A9 triggers p38 MAPK-dependent VSMC apoptosis and severe mural inflammation (120).

Epigenetic modifiers and kinase networks strictly govern this plasticity. Protectively, SIRT6 deacetylates histones to repress IL-1β transcription and prevent DNA damage (121, 122). Similarly, phosphodiesterase 5 maintains cGMP-dependent vascular tone, and its inactivation precipitates contractility loss and aneurysm progression (123). Other protective regulators include BAF60c, which maintains cellular quiescence by restraining NF-κB (124), and SAMD4A, which sustains H2AK119ub1 levels to suppress ITGB1 (125). Furthermore, adenylate kinase inhibition dampens inflammation by altering cellular methylation potential (126). In stark contrast, HMGB2 emerges as a potent pleiotropic pathogenic mediator. It provokes ROS/TLR4/NF-κB-dependent VSMC pyroptosis (127) and fundamentally warps glucose metabolism via the PPAR-γ/PGC-1α axis to stimulate aberrant proliferation (128). Concurrently, HDAC9 erases protective histone acetylation to silence contractile genes (129), ARID3A disrupts mechanosensation by repressing ATP1A2 (130), and SPSB1 drives senescence via aberrant alternative splicing (131). Moreover, mutations in the histone variant H3.3B inherently accelerate vascular disease progression (132).

Non-coding RNAs construct an additional layer of stringent regulation. Pathogenically, the long non-coding RNA Sox2ot binds miR-145 to upregulate Egr1, igniting oxidative stress (133). Similarly, SNHG16 acts as a sponge for miR-106b-5p to amplify STAT3-driven inflammation (134), and GAS5 sequesters EZH2 to derepress the RIG-I pathway, culminating in rampant interferon responses and apoptosis (135). Additionally, Rel-B drives transdifferentiation by antagonizing miR-193a-5p (136). Conversely, miR-222-3p profoundly ameliorates AD by directly silencing STAT3 (137). Other pathogenic elements include miR-485-3p, miR-335-5p, and the abnormally elevated piRNA-823, which collectively dictate synthetic transition and apoptosis (138). Furthermore, profound miR-564 downregulation removes post-transcriptional checkpoints, enabling unchecked VSMC dedifferentiation (139). Nevertheless, several microRNAs are decidedly atheroprotective. miR-146a intrinsically restricts VSMC inflammation (140), miR-126 curtails macrophage infiltration by targeting the metalloproteinase ADAM9 (141), and miR-194 abrogates KDM3A-mediated pathological demethylation (142). Ultimately, under inflammatory stress, the nuclear receptor LXRα recruits UHRF1 to hypermethylate and silence the miR-26R-3p promoter, thereby sealing the inflammatory phenotypic shift (143).

#### 3.3.3 Key signaling hubs and organelle stress

Membrane receptors, kinase cascades, and organelle stress act as critical transducers of external pathogenic stimuli. At the surface, the transporter-like protein SLC44A2 strictly governs AAA progression by modulating VSMC phenotypic switching (144). Within the ADAM family, ADAM15 confers protection by inhibiting actin depolymerization and apoptosis (145), whereas ADAM17 and ADAM9c robustly drive inflammatory cell death (141, 146). Intracellularly, the mechanistic target of rapamycin acts as a master switch for inflammatory secretion. Triggered by defective branched-chain amino acid metabolism, it prompts massive IL-6 and TNF-α release to precipitate structural failure (147, 148). Concurrently, MAPK14 integrates upstream stress to execute profound chromatin remodeling, establishing a pro-inflammatory and fibrotic matrix signature (149). Additionally, the Nox1/PAK1 axis translates Angiotensin II stimulation into ROS overproduction, directly activating MMP-2 to expand the aneurysm (150). Synergistically, MKL1 cooperates with p38 MAPK to accelerate senescence and SASP expression (151), while MAP2K2 strictly enforces phenotypic transdifferentiation via JAK/STAT activation (152). Furthermore, Aurora kinase A upregulation hyperactivates the GSK-3β/β-catenin cascade, driving the aberrant proliferation and migration that characterize vascular remodeling (153). Beyond mechanotransduction, disruption of the Keap1-Nrf2 axis abnormally triggers STING-dependent inflammation, a process effectively antagonized by paeoniflorin-mediated Nrf2 activation (152).

Crucially, endoplasmic reticulum stress bridges chronic inflammation with programmed cell death. Deficiencies in angiopoietin-like protein 8 ameliorate aneurysm progression by attenuating IRE1α/JNK-mediated endoplasmic reticulum stress; correspondingly, direct IRE1α kinase inhibition successfully aborts VSMC apoptosis (154, 155). Moreover, CARMA3 ablation provokes severe endoplasmic reticulum and mitochondrial dysfunction. This sequentially activates the NLRP3 inflammasome and GSDMD-dependent pyroptosis, releasing abundant IL-1β to aggravate AAA (156). Similarly, exogenous TNF-α triggers the PERK/eIF2α/ATF4 stress cascade to execute VSMC apoptosis (157). Finally, the cytoplasmic DNA sensor Aim2 emerges as a vital homeostatic guardian; its deletion compromises VSMC viability and accelerates calcific remodeling (158).

#### 3.3.4 Immune microenvironment and intercellular communication

Aortic pathologies parallel profound remodeling of the immune-inflammatory microenvironment (106). Stromally, connective tissue growth factor strictly preserves medial integrity. Its ablation provokes elastin fragmentation and VSMC failure, rendering the aorta critically susceptible to rupture (159). Furthermore, osteoclast-like cells secrete Sema4D to induce pathological sympathetic hyperinnervation. This triggers the neurogenic release of extracellular ATP, which activates VSMC P2rx4 receptors to drive p38 MAPK-dependent transdifferentiation (160).

Early leukocyte infiltration acts as the primary catalyst for vascular destruction. Infiltrating CD8+ T cells secrete IFN-γ and TNF-α to hyperactivate macrophages, prompting massive extrusion of MMP-2/9 and cathepsins S/K. This aggressive proteolytic cascade degrades mural elastin and directly forces VSMC dedifferentiation (161). Concurrently, BASP1+ monocytes autonomously ignite mural inflammation and propagate microenvironmental signals to force VSMC apoptosis (162). Macrophage crosstalk is particularly devastating. Macrophage GSDMD activation releases IL-1β and IL-18, dictating secondary bystander pyroptosis in adjacent VSMCs (163). Simultaneously, complement C3a stimulates VSMC-derived CCL9 production, establishing a vicious chemotactic cycle for further macrophage recruitment (164). Moreover, adventitial CX3CR1+ macrophages secrete TGF-β1 to activate the VSMC p38/SMAD2 axis, directly executing apoptosis and mural catastrophic failure (165). Crucially, macrophage-derived TNF-α silences the VSMC-specific lncRNA Carmn to permanently erase the contractile phenotype (166). Furthermore, macrophages secrete legumain to activate VSMC integrin αvβ3, dual-targeting matrix integrity and cellular survival to trigger acute dissection (167). Additionally, galectin-3 fundamentally bridges macrophage hyperactivation with VSMC apoptosis (168), while macrophage migration inhibitory factor forces VSMC autophagy and transdifferentiation via the AKT/mTOR cascade (169).

Beyond the dominant macrophage axis, endothelial- and fibroblast-derived C-type natriuretic peptide provides essential paracrine atheroprotection (170). However, in genetic aortopathies like Loeys-Dietz syndrome, CCL2+ adventitial fibroblasts act as the paradoxical primary inflammatory drivers (171). Systemically, hepatic ApoC3 aberrantly deposits within the aorta to force pathological macrophage polarization (172). Conversely, the gut microbiome metabolite indole-3-acetic acid actively curtails disease progression through potent immunometabolic modulation (173).

#### 3.3.5 Diverse forms of cell death

During AAA progression, VSMC depletion is executed through four distinct, highly regulated programmed cell death pathways. First, ferroptosis is initiated by the depletion of the protective ganglioside GM3. This derepresses ACSL4, causing lethal lipid peroxide accumulation (174). Concurrently, GPX4 downregulation cripples antioxidant clearance, synergistically ensuring ferroptotic execution (175). Second, highly pleiotropic mechanisms drive pyroptosis. Mitochondrial failure activates Caspase-3 to cleave GSDME; the resultant N-terminal fragment perforates the sarcolemma and simultaneously forces senescence and inflammatory secretion (176). Additionally, aberrant cytoplasmic IFI16 binds ASC to assemble the Caspase-1 inflammasome, precipitating severe endoplasmic reticulum stress via MCPIP upregulation (177). Furthermore, oxidative stress dismantles the Keap1-Nrf2 axis to liberate GSDMD, a pyroptotic cascade strictly reversed by targeted Nrf2 activation (178). Third, apoptosis is primarily orchestrated via mitochondrial intrinsic pathways. Extreme ROS accumulation severely skews the Bax/Bcl-2 ratio toward death. Taxifolin robustly aborts this cascade by scavenging ROS via the PI3K/Akt/Nrf2 pathway (179) and concurrently silencing TLR4/NF-κB-driven extrinsic inflammatory apoptosis (180). Finally, necroptosis mandates the intense coordinate upregulation of RIPK3 and MLKL. The potent anti-inflammatory cytokine IL-37 confers absolute protection against this necrotic demise by dismantling the TLR4/NF-κB axis to downregulate both crucial executioners (181).

While the elucidation of these highly regulated cell death pathways offers promising therapeutic targets like IL-37, critical research gaps persist in the broader context of aortopathies. A primary challenge lies in identifying the precise microenvironmental thresholds that determine whether a stressed VSMC will undergo pyroptosis, ferroptosis, apoptosis, or necroptosis. Additionally, future perspectives should focus on how to therapeutically shield the VSMC contractile apparatus from macrophage-driven proteolytic cascades including MMP-2 and MMP-9 before irreversible mural catastrophic failure occurs.

### 3.4 PAH

In PAH, pulmonary arterial smooth muscle cells (PASMCs) undergo profound pathological remodeling. Beyond hallmark hyperproliferation and apoptosis resistance, these cells adopt a pronounced immuno-inflammatory phenotype. This critical transdifferentiation is strictly governed by intrinsic molecular switches and exogenous immune cascades.

#### 3.4.1 Regulation of immune-inflammatory molecules within PASMCs

Disruption of intrinsic homeostatic networks in PASMCs strictly precipitates a pro-inflammatory state. Pathogenically, deficiency in the RNA editing enzyme ADAR1 provokes endogenous double-stranded RNA accumulation within PASMCs. Subsequent recognition by MDA5 robustly activates the MAVS-IRF3/7 cascade, unleashing a massive type I interferon response that converts PASMCs into primary inflammatory hubs, driving aggressive macrophage recruitment and vascular remodeling (182). Epigenetically, the m6A reader protein YTHDF2 is pathologically upregulated in PAH-PASMCs. It actively recognizes and stabilizes Myadm mRNA, thereby directly fueling aberrant cellular proliferation and structural remodeling (183).

Conversely, intrinsic protective pathways are frequently silenced during disease progression. The transcription factor interferon regulatory factor 7 is markedly downregulated in monocrotaline-induced pulmonary hypertension; its targeted restoration dually exerts potent anti-proliferative and anti-inflammatory effects, significantly dampening TNF-α and IL-1β expression (184). Similarly, the six-transmembrane protein STAMP2 functions as a critical mural immunosuppressor. While STAMP2 ablation does not autonomously alter PASMC proliferation, it fundamentally abolishes local immune tolerance, massively exacerbating CD68-positive macrophage perivascular infiltration and inflammatory cytokine storms (185).

#### 3.4.2 Active secretion of inflammatory mediators by PASMCs

Far from acting as passive responders, PASMCs actively sculpt the pathological immune microenvironment by secreting highly specific chemokines and adhesion molecules. Under biomechanical stimuli such as laminar shear stress, remodeling PASMCs directly upregulate and secrete CXCL12. This acts as a pivotal autocrine signal for hyperproliferation and a potent paracrine chemoattractant for immune and perivascular cells (186). Consequently, targeted neutralization of CXCL12 or its cognate receptor CXCR4 dramatically abrogates perivascular macrophage and T cell infiltration, profoundly alleviating pulmonary hypertension (187). Additionally, neural cell adhesion molecule 1 is aberrantly overexpressed in PAH-PASMCs. This molecule not only fortifies intercellular tethering to trap inflammatory cells but concurrently amplifies PASMC proliferation via the calcium/CaMKII/CREB signaling axis, thereby cementing a vicious pro-inflammatory niche (188).

#### 3.4.3 Cross-talk between PASMCs and immune cells

PAH progression heavily relies on dynamic, bidirectional crosstalk between PASMCs and diverse leukocyte populations. Recent single-cell transcriptomic analyses unveil a novel pathogenic axis driven by innate lymphocytes: the transcription factor TCF7 fuels the expansion of stress-induced natural killer cells, which directly interface with PASMCs to exacerbate pulmonary vascular disease (189).

Regarding the dominant macrophage axis, M1-polarized macrophages release miR-663b-enriched exosomes. Upon PASMC internalization, these profoundly suppress the AMPH/SIRT1 axis, forcing metabolic reprogramming and severe cellular dysfunction (190). Reciprocally, PASMCs actively recruit circulating monocytes via the CCL2/CCR2 chemotactic gradient, dictating their localized differentiation into mural macrophages (191). Furthermore, related microenvironmental shifts, intimately linked to STAMP2 signaling dysregulation, further amplify CD36-positive macrophage chemotaxis (185). Finally, adaptive immune signaling strongly synergizes with these innate responses; in Fra-2 transgenic models, type 2 cytokines, notably IL-4 and IL-13, strictly coordinate with eosinophilic infiltration to force advanced pulmonary vascular remodeling, cementing the paradigm that specific immune signals dictate PASMC transdifferentiation (192).

Although it is established that specific immune cues dictate PASMC transdifferentiation, a major current limitation is our incomplete understanding of this spatiotemporal immune specificity. The precise rules governing the dynamic recruitment of distinct immune subsets, such as innate lymphoid cells versus adaptive T cells, across different stages of PAH remain unresolved. A crucial future perspective involves exploring pharmacological strategies capable of restoring intrinsic mural immunosuppressors to effectively reverse established pulmonary vascular remodeling rather than merely halting its progression.

### 3.5 Arteritis

During the pathogenesis of arteritis, VSMCs transcend their structural roles to act as pivotal immunoregulatory and effector cells, actively orchestrating the initiation, maintenance, and structural remodeling of vascular inflammation. Crucially, arteritic VSMCs undergo a profound phenotypic shift toward a synthetic or myofibroblastic state, marked by α-SMA and CD90 expression and enhanced migratory capacity (193). In Takayasu’s arteritis, this pathological transition is fundamentally driven by the downregulation of exosomal miR-199a-5p, which unleashes MMP-2 to promote aberrant VSMC activation (194).

Furthermore, these cells exhibit profound pathological senescence, characterized by marked p16 and GL13 overexpression. Mechanistically, IL-6-induced STAT3 phosphorylation provokes mitochondrial dysfunction and impedes MFN2 degradation, generating a highly pro-inflammatory senescent phenotype (195). Consequently, VSMCs acquiring the SASP function as potent local inflammatory amplifiers. By hyper-secreting IL-6, IL-12, IL-23, and IL-1β, they directly instruct the polarization of CD4+ and CD8+ T cells toward pathogenic Th1/Tc1 and Th17/Tc17 lineages (193, 195). Concurrently, arteritic VSMCs actively dictate immune cell retention and granuloma formation by secreting a robust chemokine CXCL9, CXCL10, CXCL11, and CCL (193).

In chronic stages, a vicious bidirectional feedback loop cements the pathological microenvironment. T cell-derived IFN-γ and TNF-α chronically hyperactivate the VSMC STAT1 pathway, exponentially amplifying their pro-inflammatory output (193). Ultimately, these dysfunctional VSMCs serve as deeply entrenched local inflammatory reservoirs; even following systemic disease resolution, their persistent matrix derangements and sustained immune signaling thwart the restoration of immune tolerance, covertly driving progressive vascular stenosis and latent disease relapse (196, 197).

This phenomenon of persistent inflammatory memory highlights a critical unresolved challenge in the management of arteritis. Because these senescent VSMCs retain a senescence-associated secretory phenotype and act as latent inflammatory reservoirs despite systemic immunosuppressive therapies, future research must prioritize the identification of targeted senolytic therapies. Eradicating these persistently dysfunctional VSMCs is essential for fully restoring vascular immune tolerance and preventing covert disease relapse.

### 3.6 Cardiomyopathy and microvascular dysfunction

Beyond structural maintenance, VSMCs are fundamental to the pathogenesis of diabetic cardiomyopathy, microvascular dysfunction, and aberrant vascular remodeling. Synergistic metabolic perturbations—encompassing hyperglycemia, hypoglycemia, and advanced glycation end products (AGEs)—converge with the local immuno-inflammatory microenvironment to force VSMC phenotypic switching. This cascade ultimately dictates intimal hyperplasia, diffuse vascular calcification, and catastrophic cardiac impairment.

#### 3.6.1 Metabolic drivers of VSMC dysfunction

Metabolic toxicity acts as the primary initiator of VSMC transdifferentiation. Crucially, a hyperglycemic milieu upregulates the transcription factor Twist1. By sequestering p300, Twist1 competitively antagonizes the Myocardin/SRF complex, thereby silencing contractile genes α-SMA, SM22α and precipitating intimal hyperplasia; notably, VSMC-specific Twist1 ablation robustly aborts this diabetes-induced hyperplastic response (198). Concurrently, the pathological accumulation of AGEs vigorously induces Bcl-2-associated athanogene 3 (BAG3). BAG3 amplification directly hyperphosphorylates STAT3 to fuel excessive VSMC proliferation and migration, driving profound mural thickening and remodeling (199). Furthermore, chronic diabetic injury permanently hyperactivates calcium/calmodulin-dependent protein kinase II (CaMKII). This kinase serves as a critical nexus translating metabolic derangement into structural collapse, synchronously driving hyperproliferation and actively coordinating vascular calcification (200).

#### 3.6.2 Immune crosstalk and osteogenic transdifferentiation

The intricate crosstalk between VSMCs and innate immune cells, particularly macrophages, strictly governs vascular calcification and fibrotic remodeling. Macrophage-derived Galectin-3 emerges as a pivotal mediator; it not only actively forces the osteogenic transdifferentiation of VSMCs but also orchestrates the transmural propagation of calcification from the intima to the media, profoundly exacerbating diabetic arterial stiffness (201). Conversely, severe hypoglycemic episodes also inflict devastating vascular injury by triggering widespread endothelial apoptosis and forcing pro-inflammatory macrophage polarization. This distorted microenvironment subsequently drives VSMC fibrosis, a process putatively governed by the regulatory molecule Angptl4 (202).

In diabetic AS, Nϵ-carboxymethyllysine, a dominant AGE constituent, pathogenically downregulates the deubiquitinating enzyme USP10. This deficiency accelerates the proteasomal degradation of AMPKα, effectively erasing AMPK-dependent suppression of osteogenic genes and fiercely accelerating vascular calcification. Consequently, functional restoration of USP10 reactivates AMPK to successfully halt this calcific cascade (203).

#### 3.6.3 Microenvironmental integration in systemic stress

Under extreme systemic stress, such as sepsis-induced cardiomyopathy, the resultant immuno-inflammatory storm exerts multidimensional effects on VSMCs, with emerging evidence implicating dysregulated Dishevelled-1 signaling in this acute cellular dysfunction (204). Ultimately, the pathogenesis of DCM is not restricted to cardiomyocyte failure; it represents a comprehensive collapse of the myocardial microenvironment. Within this niche, VSMCs, alongside fibroblasts and endothelial cells, form a tightly integrated pathogenic syncytium. Accordingly, systemic interventions must target this broader cellular network. For instance, fibroblast growth factor 21 (FGF21) delivers potent cardioprotection and anti-fibrotic efficacy precisely by acting on non-cardiomyocyte populations, including the critical VSMC compartment (205).

While targeting this broader cellular network shows significant therapeutic promise, a persistent bottleneck remains in uncoupling systemic metabolic toxicity from local structural deterioration. Although the pathogenic roles of advanced glycation end products and hyperglycemia are clear, future therapeutic perspectives must focus on disrupting the precise molecular hubs that translate these systemic metabolic cues into the immune-driven osteogenic transdifferentiation of VSMCs within the integrated myocardial niche.

## 4 Clinical treatment prospects

Therapeutic interventions targeting VSMCs have advanced rapidly from superficial symptom management to profound molecular and epigenetic reprogramming. Current translational strategies primarily focus on regulating phenotypic plasticity, modulating the local immune microenvironment, accelerating the clearance of senescent or tumor-like cells, and pioneering precision nanomedicine delivery systems.>

### 4.1 Pharmacological targeting of phenotypic plasticity and calcification

Restoring the contractile VSMC phenotype and halting osteogenic transdifferentiation are primary therapeutic objectives. Current strategies employ specific pathway inhibitors and natural active compounds to interfere with metabolic switches and chromatin remodeling, forcefully reversing the synthetic phenotype and restoring cellular quiescence (**Table 1**).

**Table 1:** Interventions targeting VSMC phenotypic plasticity and vascular calcification.

Therapeutic agent/intervention	Core target/mechanism	Pathological effect/clinical application	Reference
Rapamycin	Modulates p300/CBP balance; mTOR inhibition	Reverses synthetic phenotype; enhances autophagy to clear calcification	(6, 206)
Bempedoic acid	Activates AMPK signaling pathway	Inhibits VSMC dedifferentiation in hyperlipidemia-induced vascular stiffness	(207)
Thrombospondin-1 inhibitors	Blocks leptin signaling	Attenuates VSMC pathology in obese patients	(208)
Ganoderma lucidum spore powder	Targets RUNX2-mediated osteogenic differentiation	Inhibits progressive vascular calcification	(209)
Lysyl oxidase inhibitors	Inhibits excessive LOX activity	Reduces vascular calcification	(210)
Pentamethylquercetin	Modulates PTEN/AKT/GSK-3β axis	Inhibits pathogenic phenotypic transformation	(211)
Quercetin	Inhibits JAK2/STAT3 signaling pathway	Blocks KLF4-mediated VSMC-to-macrophage transformation	(212)
Ac2-26	Mimics calmodulin-binding protein A1	Maintains VSMC homeostasis; prevents AD exacerbation	(213)

### 4.2 Regulating the inflammatory and immune microenvironment

Directly extinguishing the mural inflammatory microenvironment and interrupting pathological VSMC-immune cell crosstalk represent highly translation-viable strategies. This involves suppressing classical inflammasomes, neutralizing ROS, and utilizing natural pharmacophores to resolve vascular inflammation (**Table 2**).

**Table 2:** Therapeutic strategies modulating the VSMC immune microenvironment.

Therapeutic agent/intervention	Core target/mechanism	Pathological effect/clinical application	Reference
IL-1/NLRP3 blockade	Inhibits NLRP3 inflammasome activation	Alleviates vasculitis	(214)
PCSK9 inhibitors	Intrinsic suppression of VSMC NLRP3	Confers cardiovascular protection beyond lipid lowering	(215)
PNS R1 + Protocatechuic aldehyde	Promotes NO; blocks TGFβR1-YAP/TAZ pathway	Synergistically aborts inflammation and calcification	(216)
Physalin B	Activates Nrf2 pathway	Clears ROS to exert anti-inflammatory effects	(5)
Resveratrol	Reverses MMP expression via Nrk modulation	Protects matrix integrity	(13)
Centella asiatica acid	Inhibits NF-κB p65 and CX3CL1 pathways	Alleviates vascular remodeling	(217)
SOCS1-based mimetic peptide	Inhibits JAK and STAT pathways	Reverses severe pathological phenotypes	(218)
Paeonol	Downregulates LTβR expression	Reduces VSMC apoptosis and mural immune infiltration	(219)
Ginsenoside Rb1/IBA	Modulates autophagy; Activates LXRα	Promotes cholesterol efflux; reduces fibrosis	(220)
Pterostilbene	Modulates KEAP-1-Nrf2-STING axis	Exerts potent anti-inflammatory responses	(221)
ELABELA/Resolvin E1	Nrf2 pathway/chemR23 receptor	Suppresses oxidative stress; actively resolves inflammation	(92, 222)
Dichloroacetic acid + Atorvastatin	Synergistic metabolic/inflammatory modulation	Attenuates PAH	(223)
CCR2 blockade	Interrupts chemokine signaling	Efficacy demonstrated in PAH progression	(191)
Plant-based diet/Far-infrared radiation	Reduces TNF-α/IL-6; improves oxidative environment	Reverses microvascular dysfunction; lowers blood pressure	(224, 225)

Blocking ICAM1/ITGB2 (226) suppresses AS, but increasing CD163+ macrophages requires caution as it balances calcification inhibition with plaque vulnerability (37).

### 4.3 Senolytics and checkpoint blockade therapies

The selective eradication of senescent and pathologically hyperproliferative VSMCs offers a revolutionary therapeutic frontier. Modulating cellular lifespan and restoring immune clearance mechanisms can fundamentally halt disease progression (**Table 3**).

**Table 3:** Senolytics and proliferation-targeted interventions.

Therapeutic agent/intervention	Core target/mechanism	Pathological effect/clinical application	Reference
ABT-263	Selectively induces apoptosis in senescent cells	Dismantles pro-inflammatory SASP loop	(18, 227)
Terazosin	Stabilizes Atf3 mRNA to enhance autophagy	Delays VSMC senescence	(19)
Artemisinin	Inhibits NLRP3 inflammasome pathway	Blocks phenotypic conversion of VSMCs into macrophages	(228)
Anti-CD47 antibodies	CD47 signal	Restores macrophage phagocytosis of tumor-like clonal VSMCs in AD	(45, 229)

### 4.4 Advanced nanomedicine and precision delivery systems

Integrating potent biologics with bioengineered delivery platforms is shattering conventional pharmacokinetic limitations. Utilizing nanozymes, extracellular vesicles, and targeted nanocarriers allows for highly precise, theranostic regulation of the VSMC microenvironment (**Table 4**).

**Table 4:** Novel delivery systems and precision nanomedicine.

Therapeutic agent/intervention	Core target/mechanism	Pathological effect/clinical application	Reference
Platelet membrane-modified exosomes	Biomimetic targeting	Enables highly precise drug localization at vascular lesion sites	(230)
Polymeric nanodrugs	microRNA-146a-5p	Orchestrates targeted, post-transcriptional anti-inflammatory reversal	(231)
Bioengineered EVs	miR-145	Specifically restores the function of diseased VSMCs	(232)
Multi-functional nanozymes	CD44 receptor targeting	Aggressively scavenges ROS to halt phenotypic switching	(233)
Plaque-targeting nano-GLP-1 agonist	GLP-1 receptor	Resists degradation; integrates MRI visualization for specific differentiation tracking	(234)
Targeted Immunotherapies	TL1A cytokine; S100A4	Neutralizes specific markers on synthetic VSMCs	(235, 236)
Arterial VEGF-C delivery	Promotes lymphangiogenesis	Flushes inflammatory accumulations to stabilize atherosclerotic lesions	(237)

## 5 Conclusions

The paradigm restricting VSMCs to passive structural roles is obsolete. VSMCs are now unequivocally recognized as dynamic immunoregulatory hubs. By integrating biomechanical and metabolic stresses, they undergo profound phenotypic transitions to orchestrate the mural immune microenvironment, serving as the fundamental pathogenic engine across AS, hypertension, aneurysms, and vasculitis.

However, translating this universal pathogenic substrate into clinical efficacy faces formidable bottlenecks. Chief among them is extreme spatiotemporal heterogeneity; identical phenotypic shifts can exert diametrically opposed effects at different disease stages, as evidenced by the paradoxical dual role of IL-1β signaling discussed earlier. Furthermore, the mural interactome is immensely complex. Precisely uncoupling pathological crosstalk, such as exosomal trafficking and mitochondrial transfer, without paralyzing essential tissue repair or triggering systemic immunosuppression remains a critical translational hurdle. Crucially, current technological limitations exacerbate these challenges. Conventional single-cell sequencing necessitates tissue dissociation which intrinsically destroys the essential anatomical context, while standard *in vitro* models fundamentally fail to replicate the complex biomechanical forces and multidimensional immune cascades of the living vessel wall.

To overcome these barriers and address the specific mechanistic gaps identified in this review, precision therapeutics must aggressively prioritize three pivotal domains. First, researchers must build upon recent foundational single-cell transcriptomic studies that have successfully delineated specific VSMC-macrophage crosstalk mechanisms (118) and dynamic mesenchymal transitions in aneurysmal disease (117). Moving forward, it is imperative to integrate high-resolution spatial transcriptomics and *in vivo* lineage tracing to map a definitive atlas of VSMC subpopulations. Specifically, this mapping must decode the spatial distribution of multipotent SMC-derived intermediate cells and track how fundamental embryonic lineage heterogeneity, notably neural crest versus mesoderm origins, intrinsically dictates localized disease susceptibility. Second, to circumvent the risks of non-specific systemic immunosuppression, the field requires identifying the ultimate epigenetic and metabolic molecular switches, such as the aforementioned KLF4 or cGAS-STING axes, that irreversibly commit VSMCs to lethal, pro-inflammatory phenotypes rather than protective fibromyocyte states. This targeted knowledge will enable the engineering of stimuli-responsive, ultra-smart nanodelivery platforms designed to selectively eradicate or reprogram pathogenic VSMC subpopulations without inducing systemic off-target toxicity. Finally, it is crucial to establish multidimensional diagnostic models that integrate VSMC-specific immune activation biomarkers into routine cardiovascular risk assessments. By comprehensively deciphering the VSMC-immune interactome, we can definitively sever the root cycle of vascular damage, inaugurating a transformative era of cardiovascular precision medicine anchored in vascular immunology.
